# Psychometric validation of the revised Chinese version of the Dimensional Anhedonia Rating Scale in psychiatric outpatients

**DOI:** 10.3389/fpsyt.2026.1780405

**Published:** 2026-04-17

**Authors:** Shanshan Huang, Yuhao Wang, Hanhui Chen

**Affiliations:** Clinical Psychological Assessment Center, Tianjin Anding Hospital, Mental Health Center of Tianjin Medical University, Tianjin, China

**Keywords:** anhedonia, Chinese version, dimensional assessment, psychiatric outpatient, psychometric validation

## Abstract

**Objective:**

To refine the Chinese version of the Dimensional Anhedonia Rating Scale (DARS) and evaluate the psychometric properties of the Revised Chinese DARS (RC-DARS) in a large sample of first-visit psychiatric outpatients.

**Methods:**

The study was conducted in two sequential phases at a specialized psychiatric hospital. In Phase I (n = 277), the existing Chinese DARS underwent semantic and cultural refinement in accordance with ISPOR and TRAPD guidelines, incorporating cognitive interviews and back-translation procedures. In Phase II (n = 788), the RC-DARS was administered alongside the Self-Rating Depression Scale (SDS), Self-Rating Anxiety Scale (SAS), Pittsburgh Sleep Quality Index (PSQI), and the MMPI Suicide Ideation Subscale (MMPI-SI). Exploratory and confirmatory factor analyses were conducted using common-factor extraction and the WLSMV estimator for ordinal indicators. Internal consistency, gender-based measurement invariance, and convergent validity were evaluated.

**Results:**

Exploratory analyses supported a four-factor domain structure. Confirmatory factor analysis demonstrated good model fit for the domain-based model (χ²/df = 3.81, CFI = 0.98, TLI = 0.97, RMSEA = 0.08, SRMR = 0.05), with substantially superior fit relative to an alternative reward-processing model. Internal consistency was excellent (Cronbach’s α = 0.95; McDonald’s ω = 0.96). Multi-group analyses supported configural, metric, and scalar invariance across gender (ΔCFI < 0.01). RC-DARS total scores were significantly negatively correlated with depressive symptoms (r = −0.443), anxiety (r = −0.317), sleep disturbance (r = −0.494), and suicide risk (r = −0.312) (all p <.001). Individuals with severe depressive symptoms exhibited significantly lower RC-DARS scores than those below the clinical threshold.

**Conclusions:**

The RC-DARS demonstrates robust psychometric properties in a first-visit outpatient sample. The revision primarily enhances semantic precision and structural differentiation without materially altering score distributions. The scale may serve as a refined instrument for dimensional assessment of anhedonia in similar clinical contexts, pending longitudinal and multi-site validation.

## 1 Introduction

Anhedonia, defined as a diminished capacity to experience pleasure, is a core symptom of depressive disorders and is widely observed across multiple psychiatric conditions, including anxiety disorders, schizophrenia, eating disorders, and obsessive–compulsive disorder. (1)Within major depressive disorder, anhedonia represents a highly specific clinical feature and constitutes a diagnostic specifier in the Diagnostic and Statistical Manual of Mental Disorders, Fifth Edition (DSM-5). (2) Individuals with melancholic or anhedonic depression are characterized by profound loss of pleasure and reduced emotional reactivity to positive stimuli, particularly during acute episodes. (2).

Beyond its diagnostic relevance, anhedonia has prognostic and therapeutic implications. Anhedonic depression has been associated with seasonal patterns, increased somatic comorbidity, higher hospitalization rates, and elevated suicide risk (3–5).Although widely used for depressive disorders, conventional antidepressants such as selective serotonin reuptake inhibitors (SSRIs) and serotonin–norepinephrine reuptake inhibitors (SNRIs) may show variable and sometimes limited effects on anhedonia and reward-related dysfunction, with evidence indicating modest effect sizes and substantial heterogeneity across pharmacological mechanisms (6–9).

From a dimensional perspective, anhedonia is increasingly conceptualized as a dysfunction of reward processing (10). Within the Research Domain Criteria (RDoC) framework, anhedonia is located in the Positive Valence Systems domain, encompassing reward anticipation, motivation, consummatory pleasure, and reinforcement learning (11). Similarly, the Hierarchical Taxonomy of Psychopathology (HiTOP) model conceptualizes anhedonia as a central feature of low positive affectivity within the internalizing spectrum, linking it to depressive symptoms, anxiety, sleep disturbances, and suicidal ideation (12).

Despite its theoretical centrality, measurement remains challenging. The Snaith–Hamilton Pleasure Scale (SHAPS) primarily captures consummatory pleasure in a unidimensional format. The Temporal Experience of Pleasure Scale (TEPS) differentiates anticipatory and consummatory pleasure but lacks domain anchoring (13). The DARS adopts a domain-based approach, assessing non-social, food-related, social, and sensory pleasure, while integrating affective enjoyment with motivational and effort-related components (14). This domain-anchored format reduces reliance on culturally specific exemplars and permits personally relevant contextualization within each domain, making it particularly advantageous for cross-cultural adaptation.

Although a Chinese version of the DARS has previously been reported and demonstrated acceptable reliability and validity, several issues remain relevant for clinical application (15). In particular, during routine administration in outpatient settings, some items were found to contain minor semantic ambiguities and potential overlap between motivational engagement and affective enjoyment, which may reduce conceptual differentiation among hedonic components. These concerns do not necessarily indicate psychometric inadequacy of the existing scale but rather highlight opportunities for linguistic refinement and improved conceptual clarity in cross-cultural adaptation.

Cross-cultural scale adaptation guidelines emphasize that translation is an iterative process requiring ongoing evaluation of semantic precision, cultural relevance, and construct equivalence beyond initial validation. Therefore, the present study aimed to conduct a systematic semantic and cultural refinement of the Chinese DARS following established ISPOR and TRAPD guidelines and to evaluate the psychometric properties of the Revised Chinese version of the DARS (RC-DARS) in a large sample of first-visit psychiatric outpatients (16, 17).

## 2 Methods

### 2.1 Participants

The study was conducted in two sequential phases at a specialized psychiatric hospital in Tianjin, China. Phase I included 300 consecutively recruited first-visit psychiatric outpatients for preliminary testing and item refinement; 277 valid cases remained after data quality control. Phase II included 800 consecutively recruited first-visit outpatients; 788 valid cases were retained.

Inclusion criteria were: (1) first-time presentation to the hospital and (2) prominent symptoms of anxiety and/or depression. Exclusion criteria included organic brain disorders and overt psychotic symptoms such as hallucinations, delusions, or hypomanic episodes.

Formal diagnostic information was available only for a subset of participants due to the constraints of routine outpatient practice and ethical considerations. Diagnostic data were derived from consecutively recorded clinical diagnoses documented in the electronic medical record system. The subsamples (n = 100 in Phase I and n = 200 in Phase II) consisted of all participants for whom diagnostic evaluations had been completed and recorded at the time of data extraction, without any additional selection procedures.

### 2.2 Measures

The DARS assesses hedonic capacity across four activity domains. Higher scores indicate greater pleasure capacity and lower anhedonia severity.

Phase I participants completed the previously published Chinese DARS (C-DARS). Phase II participants completed the Revised Chinese DARS (RC-DARS). Additional measures included Self-Rating Depression Scale (SDS), Self-Rating Anxiety Scale (SAS), Pittsburgh Sleep Quality Index (PSQI), and MMPI Suicide Ideation Subscale (MMPI-SI) (18–21).

Item-level revisions and rationales are detailed in **Appendix S1**.

### 2.3 Procedure and scale revision

The revision process followed ISPOR Good Practice Guidelines and the TRAPD model (Translation, Review, Adjudication, Pretesting, Documentation) (16, 17). Fifty Phase I participants were selected using computer-generated random numbers from the full Phase I dataset for cognitive interviews assessing clarity, semantic accuracy, cultural appropriateness, and differentiation between motivational and affective components. Demographic characteristics of this subset did not differ significantly from the overall Phase I sample, suggesting that the subset was broadly representative. Items demonstrating ambiguity or motivational–affective conflation were revised. All revised items underwent back-translation and conceptual equivalence verification.

### 2.4 Statistical analysis

All statistical analyses were conducted using SPSS 22.0 and Mplus 8.3. Prior to exploratory factor analysis (EFA), sampling adequacy was evaluated using the Kaiser–Meyer–Olkin (KMO) measure and Bartlett’s test of sphericity, together with inspection of eigenvalues and the scree plot. EFA was performed using principal axis factoring with Promax (oblique) rotation. The number of factors retained was determined based on the theoretical structure of the original DARS, eigenvalue magnitude, scree plot examination, and the interpretability and conceptual coherence of the factor solution.

For confirmatory factor analysis (CFA), Phase II data (N = 788) were randomly divided into two equal subsamples (n = 394 each) using computerized randomization. Subsample A was used to replicate the exploratory structure, and Subsample B was used for CFA. CFA was conducted with the weighted least squares mean and variance adjusted (WLSMV) estimator, which is appropriate for ordinal indicators. Model fit was evaluated using χ²/df, the comparative fit index (CFI), the Tucker–Lewis index (TLI), the root mean square error of approximation (RMSEA), and the standardized root mean square residual (SRMR).

Internal consistency reliability was assessed using Cronbach’s α, McDonald’s ω, and corrected item–total correlations. Measurement invariance across gender was examined via multi-group CFA, sequentially testing configural invariance, metric invariance (equality of factor loadings), and scalar invariance (equality of item thresholds). Invariance was considered supported when changes in fit indices met established criteria (|ΔCFI| ≤ 0.01 and ΔRMSEA ≤ 0.015).

Convergent validity was evaluated using Pearson correlation coefficients between RC-DARS scores and clinical symptom measures. All statistical tests were two-tailed, with p < 0.05 considered statistically significant.

## 3 Results

### 3.1 Sample characteristics

The final sample sizes were 277 participants in Phase I and 788 participants in Phase II. **Table 1** presents the demographic characteristics of the samples before and after revision. Significant differences were observed between the two phases in gender distribution, educational level, and employment status (all p <.05), whereas no significant differences were found in age or marital status.

**Table 1:** Demographic characteristics of participants pre- and post-revision of the Chinese DARS.

Characteristic	Pre-revision (n=277)		Post-revision (n=788)		*χ²*	*df*	*p*
	n	%	n	%			
Gender					5.63	1	0.02
Female	185	66.79	469	59.52			
Male	92	33.21	319	40.48			
Age group (years)					2.43	3	0.48
≤20	51	18.41	123	15.55			
21-40	128	46.21	403	51.16			
41-60	71	25.63	184	23.39			
≥61	27	9.75	78	9.90			
Educational level					12.05	3	<0.01
Master’s degree or above	19	6.86	44	5.58			
College/Undergraduate	155	55.96	488	61.93			
High school	33	11.91	125	15.86			
Middle school or below	70	25.27	131	16.62			
Employment status					21.61	1	<0.01
Unemployed	109	39.35	197	24.94			
Employed	168	60.65	591	75.06			
Marital status					2.40	2	0.30
Unmarried	111	40.07	300	38.07			
Married	142	51.26	393	49.87			
Divorced/Widowed	24	8.66	95	12.06			

Regarding clinical characteristics, comprehensive diagnostic data were not available for all participants due to constraints inherent in routine outpatient practice and ethical considerations. Therefore, first-visit diagnostic information was extracted for subsets of participants, including 100 individuals from Phase I and 200 individuals from Phase II. In Phase I (n = 100), diagnoses included obsessive–compulsive disorder (n = 1), major depressive disorder (n = 19), bipolar disorder (n = 33), anxiety disorders (n = 17), and other or no definite diagnosis (n = 30). In Phase II (n = 200), diagnoses included obsessive–compulsive disorder (n = 6), major depressive disorder (n = 12), bipolar disorder (n = 67), anxiety disorders (n = 38), and other or no definite diagnosis (n = 77). A comparison of diagnostic distributions between the two subsets revealed a significant difference (χ² = 13.55, p = .01), indicating that the two samples were not fully equivalent in clinical composition. This potential imbalance is acknowledged and discussed as a limitation.

### 3.2 Score stability

No significant differences were observed between pre-revision and post-revision samples in total DARS scores or any subscale scores (all p > 0.05), indicating semantic refinements did not materially alter score distributions (**Table 2**).

**Table 2:** Comparison of dimension scores and total scores of the Chinese DARS pre- vs. post-revision.

Score	Pre-revision (n=277)	Post-revision (n=788)	t (p)
Hobbies	9.1 (3.9)	8.9 (4.1)	0.91 (0.37)
Food/drink	8.6 (3.8)	8.7 (3.9)	-0.53 (0.59)
Social activities	8.0 (4.3)	7.5 (4.4)	1.42 (0.16)
Sensory Experience	10.7 (5.2)	10.5 (5.3)	0.38 (0.70)
Total score	36.3 (14.6)	35.7 (14.8)	0.66 (0.51)

### 3.3 Exploratory factor analysis

Exploratory factor analyses were conducted separately for the pre-revision and post-revision samples. Sampling adequacy was excellent in both datasets, with Kaiser–Meyer–Olkin (KMO) values of 0.94 in each sample. Bartlett’s test of sphericity was significant for both the pre-revision (χ² = 3743.16, df = 136, p <.001) and post-revision samples (χ² = 10825.60, df = 136, p <.001), supporting the suitability of the correlation matrices for factor analysis.

In the pre-revision sample, the first four eigenvalues were 9.30, 1.58, 1.24, and 0.94, accounting for 76.8% of the total variance. In the post-revision sample, the corresponding eigenvalues were 9.07, 1.68, 1.24, and 0.99, accounting for 76.3% of the variance. Although an additional factor initially emerged during extraction, the fifth factor exhibited weak loadings (<.50), notable cross-loadings, and lacked conceptual coherence. Accordingly, based on eigenvalue magnitude, scree plot inspection, factor interpretability, and alignment with the theoretical structure of the original DARS, a four-factor solution was retained in both samples.

Notably, in the pre-revision sample, item loadings were disproportionately concentrated on the first two factors, indicating insufficient differentiation among the theoretically specified domains (**Table 3**). In contrast, the post-revision solution demonstrated clearer factor separation and improved alignment with the intended multidimensional structure (**Table 4**).

**Table 3:** Factor loadings of the four-factor model for the pre-revision Chinese DARS.

Item	Factor			
	1	2	3	4
1	.896	-.050	.025	-.060
3	.860	-.015	-.006	.012
2	.820	.076	-.008	-.025
4	.795	.017	.028	.055
15	-.013	.894	-.065	-.042
16	.091	.787	-.091	.086
17	-.062	.784	.117	.027
14	-.027	.716	.175	-.010
13	.088	.676	.125	.022
10	.028	-.154	.975	.053
12	-.045	.152	.811	-.031
11	-.006	.177	.696	-.053
9	.096	.042	.662	.117
7	.011	-.100	-.015	.983
6	.097	.161	-.108	.732
8	-.103	.005	.091	.704
5	.000	.059	.108	.594

**Table 4:** Factor loadings of the four-factor model for the Revised Chinese version of the DARS.

Item	Factor			
	Sensory experience	Hobbies	Social activities	Food/drink
16	.955	.030	-.150	.005
15	.868	.006	-.002	-.034
17	.741	-.073	.143	.021
14	.726	.042	.118	.002
13	.627	.017	.195	.052
2	-.036	.931	.008	-.068
3	-.037	.837	.005	.057
1	.036	.831	-.032	-.035
4	.086	.677	.065	.089
10	-.097	.009	.955	.008
12	.076	-.019	.847	-.041
9	.050	.089	.717	.046
11	.107	-.037	.701	.033
7	.029	-.049	-.064	.948
8	-.025	-.023	.052	.724
6	.028	.166	-.064	.721
5	-.018	-.041	.127	.663

### 3.4 Confirmatory factor analysis

**Figure 1:**
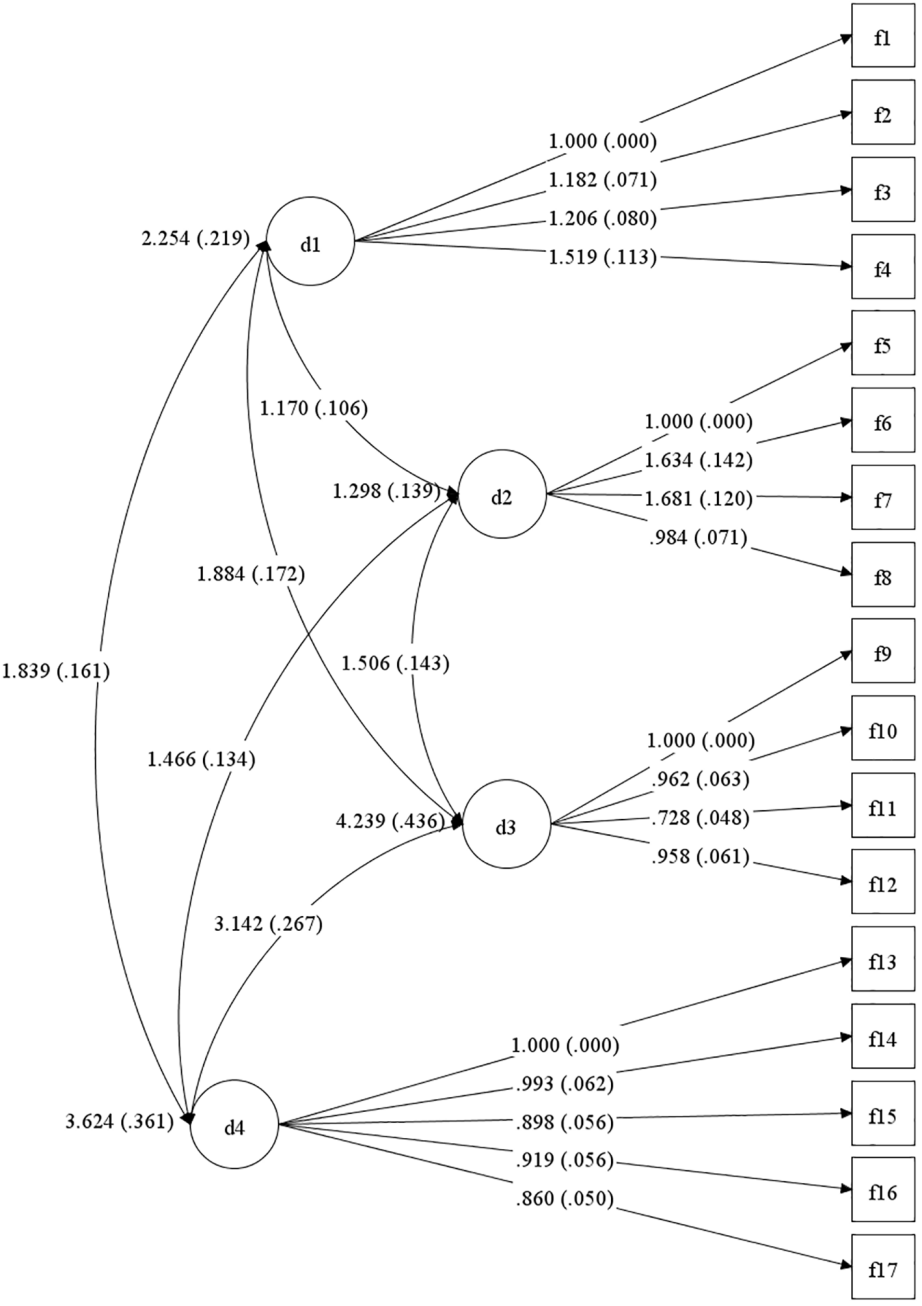
Domain-based factor structure of the Revised Chinese version of the DARS.The numbers in brackets in the figure represent standardized regression coefficients. d1, Please list at least 2 of your favorite pastimes/hobbies that are NOT primarily social; f1, I would enjoy these activities; f2, I would spend time doing these activities; f3, I want to do these activities; f4, These activities would interest me; d2, Please list at least 2 of your favorite foods or drinks; f5, I would make an effort to get/make these foods/drinks; f6, I would enjoy these foods/drinks; f7, I want to have these foods/drinks; f8, I would eat as much of these foods as I could; d3, Please list at least 2 of your favorite social activities; f9, Spending time doing these things would make me happy; f10, I would be interested in doing things that involve other people; f11, I would be the one to plan these activities; f12, I would actively participate in these social activities; d4, Please list at least 2 of your favorite sensory experiences; f13, I would actively seek out these experiences; f14, I get excited thinking about these experiences; f15, If I were to have these experiences I would savor every moment; f16, I want to have these experiences; f17, I would make an effort to spend time having these experiences.

Confirmatory factor analysis (CFA) was conducted on the post-revision sample using the WLSMV estimator appropriate for ordinal data. The domain-based four-factor model demonstrated good model fit (χ²/df = 3.81, CFI = 0.98, TLI = 0.97, RMSEA = 0.08, SRMR = 0.05, **Figure 1**), whereas the alternative reward-processing-based model showed substantially poorer fit (χ²/df = 38.81, CFI = 0.79, TLI = 0.69, RMSEA = 0.16). All standardized factor loadings in the four-factor model were statistically significant (p <.001), no Heywood cases were observed, and the standardized factor correlations ranged from 0.11 to 0.28, indicating moderate associations without evidence of redundancy. Together, these results support the adequacy and structural validity of the domain-based four-factor model over the competing reward-processing framework.

### 3.5 Reliability

The revised RC-DARS demonstrated excellent internal consistency, with Cronbach’s α = 0.95 and McDonald’s ω = 0.96. All item–total correlations exceeded.50, and deletion of any individual item did not materially improve reliability, indicating strong item homogeneity without evidence of a problematic item. Split-half reliability coefficients were 0.90 for non-social pleasure, 0.88 for food-related pleasure, 0.92 for social pleasure, and 0.81 for sensory pleasure; corresponding coefficients for anticipated pleasure, consummatory pleasure, effort-related pleasure, and motivational pleasure were 0.91, 0.89, 0.85, and 0.88, respectively, further supporting the stability and internal coherence of the scale across domains.

### 3.6 Measurement invariance

Measurement invariance across gender was evaluated using a sequential multi-group CFA framework. The metric invariance model (CFI = 0.983, RMSEA = 0.062) did not show meaningful deterioration in fit relative to the configural model (CFI = 0.976, RMSEA = 0.060), with minimal changes in fit indices (ΔCFI = −0.007, ΔRMSEA = 0.002). These results support the equivalence of factor loadings across male and female participants.

The scalar invariance model (CFI = 0.968, RMSEA = 0.062), which additionally constrained item thresholds to equality across gender, also demonstrated acceptable fit. Changes relative to the metric model were small (ΔCFI = −0.008, ΔRMSEA = 0.002) and remained within recommended cutoffs.

Taken together, the findings support scalar invariance of the RC-DARS across gender, indicating that the factor structure, loadings, and item thresholds are comparable between male and female patients. Accordingly, comparisons of latent mean differences across gender can be interpreted with appropriate methodological confidence.

### 3.7 Associations with clinical symptoms

RC-DARS total scores were significantly negatively correlated with depressive symptoms (SDS, r = −0.443), anxiety symptoms (SAS, r = −0.317), sleep disturbance (PSQI, r = −0.494), and suicide risk (MMPI-SI, r = −0.312), with all correlations reaching statistical significance (p <.001) (**Table 5**). These findings indicate that lower hedonic capacity was associated with greater overall clinical severity. Given the cross-sectional design, regression analyses were not performed in order to avoid overinterpretation of directional or predictive relationships. .

**Table 5:** Correlations between RC-DARS scores and other clinical measures scores.

	RC-DARS	SDS	SAS	PSQI	MMPI.SUCIDE
RC-DARS	1				
SDS	-.443**	1			
SAS	-.317**	.625**	1		
PSQI	-.494**	.522**	.222	1	
MMPI.SUCIDE	-.312**	.496**	.383**	.277	1

RC-DARS, Revised Chinese version of the Dimensional Anhedonia Rating Scale; SDS, Self-Rating Depression Scale; SAS, Self-Rating Anxiety Scale; PSQI, Pittsburgh Sleep Quality Index; MMPI–Suicide, Suicide Ideation Subscale of the Minnesota Multiphasic Personality Inventory.

One-way analysis of variance (ANOVA) further demonstrated that participants meeting the SDS cutoff for depression (SDS ≥ 63, n = 410) scored significantly lower on the RC-DARS total scale and across all domain subscales compared with those below the cutoff (SDS < 63; see **Table 6**), supporting the scale’s discriminative validity within depressive symptom spectra.

**Table 6:** Differences in RC-DARS scores across clinical subgroups.

Domains	Depression (n=410/378)		Anxiety (n=512/276)	
	F	p	F	p
Hobbies	7.61	<0.01	1.42	0.23
Food/drink	0.89	0.34	1.21	0.24
Social activities	8.21	<0.01	1.87	0.17
Sensory Experience	7.58	<0.01	3.92	0.49
Total scores	8.04	<0.01	2.15	0.14

In contrast, when stratified by anxiety severity using the SAS cutoff (SAS ≥ 60, n = 512 vs. SAS < 60), no statistically significant differences were observed in RC-DARS total or domain scores between the high- and low-anxiety groups. This pattern suggests that, although RC-DARS scores are moderately correlated with continuous anxiety symptom levels, group-based discrimination using the conventional SAS threshold may be less pronounced in this sample.

## 4 Discussion

This study provides cross-sectional psychometric validation of the Revised Chinese DARS (RC-DARS) in a large sample of first-visit psychiatric outpatients. Following systematic semantic and cultural refinement, score distributions remained stable between pre- and post-revision samples, while factor analytic results indicated improved structural differentiation in the revised version. Exploratory analyses in both phases supported a four-factor solution consistent with the original domain-based structure; however, the pre-revision version showed suboptimal factor separation, whereas the post-revision solution demonstrated clearer alignment with the intended multidimensional domains. Confirmatory factor analysis using an ordinal estimator further supported the adequacy of the four-factor domain model, which showed substantially better fit than an alternative reward-processing model. Internal consistency was high (α = 0.95, ω = 0.96), with strong item–total correlations and no evidence of problematic items, and multi-group analyses supported configural, metric, and scalar invariance across gender. RC-DARS scores were moderately and negatively associated with depressive, anxiety, sleep, and suicide-related symptoms, and group comparisons indicated lower hedonic capacity among individuals with depressive disorders, supporting convergent and discriminative validity within the affective spectrum.

The findings of the present study should be interpreted within the context of several limitations. First, the sample was derived from a single-site, symptom-defined outpatient population, and formal diagnostic data were available only for a subset of participants, with some imbalance in diagnostic composition across phases. Second, all measures were based on self-report, which may introduce common method variance. Third, the cross-sectional design precludes conclusions regarding temporal stability, sensitivity to change, or predictive validity.

Importantly, the present revision should be understood as a methodological refinement rather than a fundamental redevelopment of the instrument. While the RC-DARS demonstrated improved structural differentiation and clearer alignment with the intended domain-based framework, score distributions remained largely comparable to those of the original version, suggesting that the differences are primarily related to semantic precision and construct clarity rather than substantial changes in measured clinical severity.

Future research should extend these findings by examining test–retest reliability, longitudinal sensitivity to clinical change, and measurement invariance across diagnostic groups and cultural contexts. Multi-site studies incorporating clinician-rated and behavioral indicators of reward processing would further strengthen the evidence base and clarify the incremental utility of the revised instrument in both research and clinical applications.

## 5 Conclusion

The RC-DARS demonstrates strong internal consistency, a stable factorial structure, and theoretically coherent associations with affective symptom measures in a first-visit psychiatric outpatient sample. The present findings suggest that the revision primarily enhances semantic precision and structural differentiation, while overall score distributions remain comparable to those of the original version. Accordingly, the RC-DARS may serve as a refined instrument for the dimensional assessment of anhedonia in similar clinical contexts. However, its incremental utility and generalizability require further evaluation through longitudinal designs, multi-site samples, and the inclusion of clinician-rated and behavioral measures.

## Data Availability

The raw data supporting the conclusions of this article will be made available by the authors, without undue reservation.
