# Supplementary material for: Psychometric validation of the revised Chinese version of the Dimensional Anhedonia Rating Scale in psychiatric outpatients
Source: Front Psychiatry. 2026 Apr 17;17:1780405. doi: 10.3389/fpsyt.2026.1780405 (PMC13133005; doi:10.3389/fpsyt.2026.1780405)
Supplement: Supplementary file 1 [file SupplementaryFile1.docx]

# Appendix S1. Comparison of Original and Revised Chinese Translations with English Text of the Dimensional Anhedonia Rating Scale (DARS)

| **Item** | **English Text** | **Original Chinese Translation** | **Revised Chinese Translation** |
| --- | --- | --- | --- |
| d1 | Please list at least 2 of your favorite pastimes/hobbies that are NOT primarily social | 请列举至少两种您最喜欢的非社交性的娱乐活动/爱好 | 请选择两种或以上您最喜欢的非社交兴趣爱好 |
| f1 | I would enjoy these activities | 我喜欢享受这些事情 | 我会享受这些活动 |
| f2 | I would spend time doing these activities | 我愿意花时间去做这些事情 | 我愿意花时间去做这些活动 |
| f3 | I want to do these activities | 我想去做这些事情 | 我想去做这些活动 |
| f4 | These activities would interest me | 这些事情让我感兴趣 | 这些活动会让我感兴趣 |
| d2 | Please list at least 2 of your favorite foods or drinks | 请列举出至少两种您最喜欢的食物/饮料 | 请选择两种或以上您最喜欢的食物/饮品 |
| f5 | I would make an effort to get/make these foods/drinks | 我会努力得到/制作这些食物/饮料 | 我会努力获取/制作这些食物/饮品 |
| f6 | I would enjoy these foods/drinks | 我喜欢这些食物/饮料 | 我会享受这些食物/饮品 |
| f7 | I want to have these foods/drinks | 我想要拥有这些食物/饮料 | 我想要吃这些食物/喝这些饮品 |
| f8 | I would eat as much of these foods as I could | 我会尽量多吃这些食物 | 我会尽量多地吃这些食物/喝这些饮品 |
| d3 | Please list at least 2 of your favorite social activities | 请列举出至少两种您最喜欢的社会活动 | 请选择两种或以上您最喜欢的社交活动 |
| f9 | Spending time doing these things would make me happy | 花时间做这些事情能让我感到快乐 | 花时间做这些活动会让我感到快乐 |
| f10 | I would be interested in doing things that involve other people | 我乐意和他人一起做这些事情 | 我愿意和他人一起做这些活动 |
| f11 | I would be the one to plan these activities | 我愿意来筹划这些事情 | 我愿意成为组织安排这些活动的人 |
| f12 | I would actively participate in these social activities | 我将积极参与这些社会活动 | 我会积极参与这些社会活动 |
| d4 | Please list at least 2 of your favorite sensory experiences | 请列举出至少两项您最喜欢的感觉体验 | 请选择两种或以上您最喜欢的感官体验 |
| f13 | I would actively seek out these experiences | 我愿意积极寻求这些体验 | 我会积极寻求这些体验 |
| f14 | I get excited thinking about these experiences | 想到这些体验能让我感到兴奋 | 想到这些体验会让我感到兴奋 |
| f15 | If I were to have these experiences I would savor every moment | 如果我将经历这些体验，我会细细品味每一刻 | 如果我能经历这种体验，我会好好享受每一刻 |
| f16 | I want to have these experiences | 我想拥有这些体验 | 我想要拥有这些体验 |
| f17 | I would make an effort to spend time having these experiences | 我将努力花时间去拥有这些体验 | 我会努力花时间去拥有这些体验 |

*Note: The revised Chinese items enhance linguistic fluency and semantic precision, minimize cognitive load, and explicitly differentiate anticipatory motivation, effort allocation, and sense of agency by optimizing modality, tense, and culturally appropriate phrasing, thereby improving conceptual clarity and construct validity.*
