# Supplementary material for: Psychometric validation of the revised Chinese version of the Dimensional Anhedonia Rating Scale in psychiatric outpatients
Source: Front Psychiatry. 2026 Apr 17;17:1780405. doi: 10.3389/fpsyt.2026.1780405 (PMC13133005; doi:10.3389/fpsyt.2026.1780405)
Supplement: Supplementary file 2 [file SupplementaryFile2.docx]

# Appendix S2. Scree plots for Exploratory Factor Analysis of the Pre- vs. Post- Revision


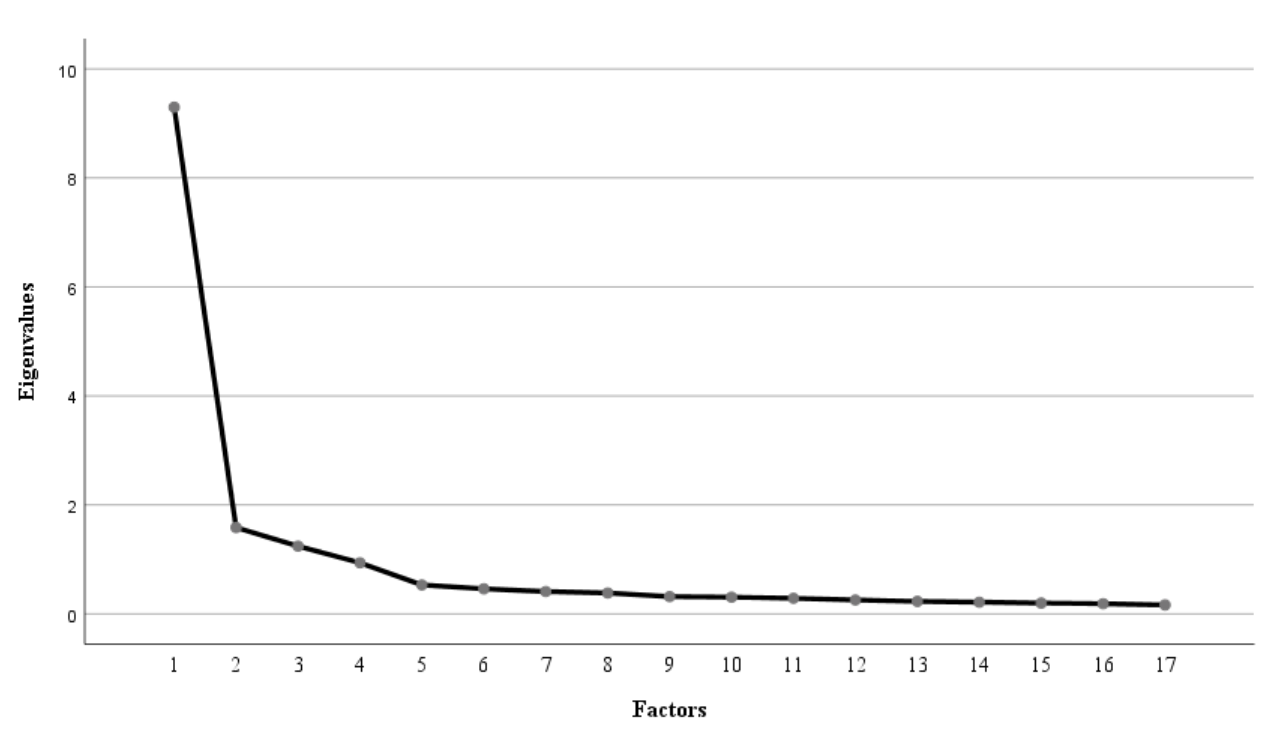


Pre-Revision


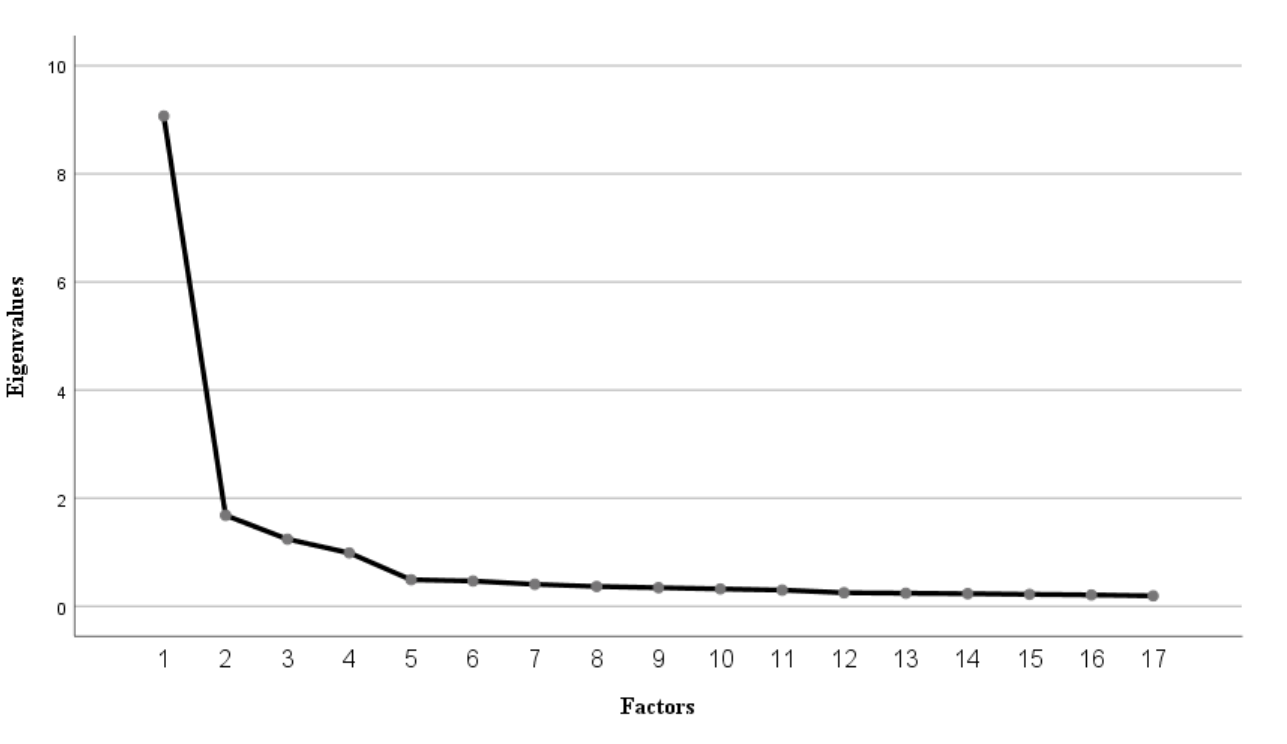
Post-Revision
